# Unraveling the causal influences of drought and crop production on groundwater levels across the contiguous United States

**DOI:** 10.1093/pnasnexus/pgaf129

**Published:** 2025-04-28

**Authors:** Nitin K Singh, Sheila M Saia, Ruchi Bhattacharya, Hoori Ajami, David M Borrok

**Affiliations:** Department of Crop, Soil, and Environmental Sciences, Auburn University, Auburn, AL 36849, USA; Center for Ecological Analytics and Modeling, Tetra Tech, Research Triangle Park, Durham, NC 27703, USA; Biological, Geological and Environmental Sciences, Cleveland State University, Cleveland, OH 44115, USA; Department of Environmental Sciences, University of California Riverside, Riverside, CA 92521, USA; College of Engineering and Computing, Missouri University of Science and Technology, Rolla, MO 65409, USA

**Keywords:** water sustainability, food security, groundwater decline, causality modeling, drought, Environmental Sciences (Physical Sciences and Engineering)

## Abstract

Groundwater depletion in agricultural-dominated regions is attributed to climate and irrigation withdrawals that support crop production. However, despite decades of effort, knowledge gaps remain in understanding the relative influence of drought and crop production on groundwater levels at the continental scale. Here, utilizing empirical observations, we simultaneously track how long-term trajectories of groundwater levels, crop production of seven crops, and drought have evolved over time, and then integrate these observations with a causality-based attribution framework to unravel the relative impact of drought and crop production on groundwater levels across the contiguous United States (CONUS). We find a dominant pattern of decreases in groundwater levels with increases (25–61%) or no change (1–15%) in crop production across the CONUS. We estimate a significant (*P* < 0.1) causal influence of crop production and drought on groundwater levels in ∼32% (*n* = 101) and ∼20% (*n* = 62) of counties, respectively. Further, the extent of impact of crop production on groundwater varies with region and is most pronounced for cotton (42%, *n* = 18) and wheat (17%, *n* = 39). The memory effects of crop production (median: 7 years) and drought (median: 3 years) on groundwater levels imply that their impact could last much longer than the annual crop production cycle or the drought exposure period. Further, these findings allude to circular causality between groundwater and crop production, where both entities depend on each other at different time scales. Our work builds on past work and contributes to the growing understanding of food security and groundwater availability to manage these commodities to meet future demands.

**Significance Statement**

Sustainable groundwater management is a global concern due to frequent droughts and increasing demand for food production. However, the relative impact of drought and crop production on groundwater levels remains unclear. Crop production may have a greater influence than drought on groundwater levels for the study counties. Further, the impact of drought and crop production on groundwater levels can last much longer than the annual crop production cycle and drought exposure period. We further highlight some vulnerable agricultural regions that may require interventions to mitigate the impact of drought and management practices on groundwater levels in the United States. Our work emphasizes the need for effective strategies to manage these two competing commodities and achieve future food and water security goals.

## Introduction

Groundwater levels continue to decline rapidly across the United States and globally (1–6). Many aquifer systems have shown a decline in groundwater level, attributable in part to changing climatic conditions (e.g. drought) and increased groundwater withdrawal for irrigation to sustain crop production (7–11). Despite decades of effort in driver attribution of groundwater, knowledge gaps remain to highlight the relative impact of drought and crop-specific stress owing to irrigation on groundwater levels at a continental scale.

To quantify the impact of crop production on groundwater levels, most studies have used hydrological models and satellite datasets to simulate water budgets and associated irrigation-driven groundwater withdrawal (7, 12–14). A subset of these modeling studies has linked groundwater usage to specific crop types (13–15). Such studies rely on regional to continental scales hydrological models that simplify the hydrologic system and have many parameters to simulate the relationship between each crop and groundwater (16). Others have used a combination of crop or statistical models to establish empirical relationships between groundwater and the production of specific crops (17–20). The major impediment in understanding the crop-specific influence on groundwater level is the lack of adequate long-term time series data for irrigation at a high spatiotemporal resolution (21, 22). Thus, there remains a need to understand how the production of specific crops can impact groundwater levels while using empirical observations over the long term at a fine resolution across the contiguous United States (CONUS).

Climate affects groundwater levels directly and indirectly (23). Hence, many studies in the United States and elsewhere have attributed the changes in groundwater levels to climatic variables (such as precipitation anomalies and drought) by often analyzing groundwater level data from heavily managed to pristine landscapes (9, 24–27). Due to the rise in the frequency of droughts globally, quantifying the impact of drought on groundwater levels has become of utmost importance for better groundwater management under changing climatic conditions. Several studies have used empirical observations to explore the impact of drought on groundwater levels (28, 29). However, these studies relied on correlation between groundwater levels and drought indices (e.g. standard precipitation index) to quantify the impact of drought on groundwater levels. The use of correlation in this context poses a few challenges. The drought propagation from meteorological forcings to hydrologic systems (e.g. groundwater) follows a cause-effect relationship (30). However, correlation can be coincidental, and simply the presence of a significant correlation between two variables does not mean causation (10, 31, 32). Hence, there is a need to utilize a cause-effect (i.e. causality) based approach to investigate the impact of drought on groundwater levels across CONUS. The causality-based framework is considered more robust and is widely used in climate change detection and attribution studies, where results often face public and political scrutiny and litigation (33).

To address these knowledge gaps, we utilize a robust, causality-based attribution approach to test the hypothesis that—crop production or drought can “cause” groundwater level changes within each county. Testing this hypothesis will allow us to reveal the relative impact of drought and the production on groundwater levels at a county scale across CONUS. Collectively, the select seven field crops represent ∼60% of the total acreage area based on long-term annual means, have some of the highest irrigation water usage (14) in the United States and contributes substantially to national and international food chain (15, 34, 35). First, we examine county scale, long-term trajectories of groundwater levels, drought indices (standard precipitation index, palmer hydrological drought index [PHDI]), and crop production for the key crops (wheat, corn, cotton, rice, barley, oat, soybean) in the United States. Second, we use causality to highlight the relative role of crop production and drought in mediating groundwater levels. The motivating research questions we address are: (i) what are the historical trends in groundwater levels, drought, and crop production for these seven crops within groundwater-irrigation-dominated counties (∼550) during the past four decades? and (ii) to what extent do crop production or drought “cause” groundwater level change across these agricultural regions?

## Results and discussion

### Trends in groundwater, crop production, and drought

**Fig. 1.**
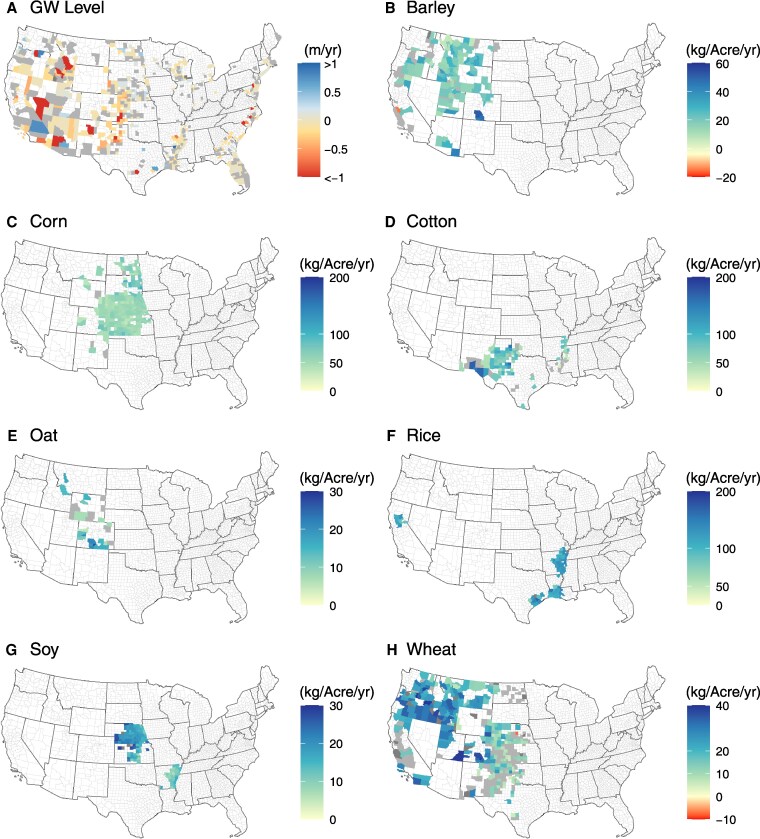
Temporal trends in annual median groundwater levels (A) and annual irrigated crop production for barley (B), corn (C), cotton (D), oat (E), rice (F), soybean (G), and wheat (H) across groundwater-irrigation-dominated counties during 1970–2018. The color gradients represent the rate of change (i.e. sen slopes), and gray-colored polygons represent no significant (*P* > 0.1) change in county-scale trends.

Trend analysis of median annual groundwater levels shows a significant (*P* < 0.1) decline in 41% of counties, a significant (*P* < 0.1) increase in 21% of counties, and no significant trend (*P* > 0.1) in 38% of counties during a 49 years (1970–2018) period (Fig. 1). On average, the rate of decline is highest in groundwater-irrigation-dominated counties of the western United States such as Sandoval, New Mexico (median rate: 3.6 m/year). At the scale of relevant aquifers, the steep declines in groundwater levels are noted in the Basin and Range carbonate-rock aquifer in Nevada region (median: 2.88 m/year), Texas coastal uplands and Columbia Plateau basin-fill aquifer (median: ∼1.8 m/year). These results are consistent with the recent studies documenting trends in groundwater level and storage across CONUS (9, 36).

We find that most crops show significant (*P* < 0.1) upward trends in production in more than 60% of counties over the last four decades (Fig. 1). Corn and rice show increasing trends in crop production for more than 90% of counties (Fig. 1). The rate of increase is the greatest for corn (median: 55 kg/acre/year) and the lowest for cotton (median: 8 kg/acre/year). Crops show a varied degree of stagnation in production; 1–37% of counties demonstrate nonsignificant trends in crop production (Fig. 1). Previously, a study highlighted the stagnation in select crops such as rice, corn, soybean, and wheat globally from 1961 to 2008 (37). Here, however, we show that the extent of stagnation in crop production is present, to varying degrees, across seven crops at the county scale in the United States. While these findings highlight the need for efficient agriculture management practices, opportunities remain to explore other potential economic and social drivers to alleviate crop yield stagnation.

We compare temporal trends between crop production and groundwater levels in ∼300 counties where both crop production and groundwater data are available (Fig. S1). A significant (*P* < 0.1) decline in groundwater levels and a significant (*P* < 0.1) increase in crop production is one of the dominant patterns (25–60% counties; *n* = 5–57) across crops in the United States (Fig. S1). We also note no significant change in crop production but a significant decrease in groundwater levels for cotton, corn, barley, wheat, and oat (1–15% counties; *n* = 1–34). Interestingly, several of these counties are in the Central High Plains aquifer (i.e. Colorado, Kansas, Nebraska, New Mexico, Oklahoma, South Dakota, Texas, and Wyoming) where groundwater supplies are currently stressed (Fig. 1A). It is likely that groundwater availability may not be adequate for wheat production, the region's dominant crop, in the years to come. These results align with a recent study that projected a decline in wheat production by 50% due to declining groundwater levels and recommends switching to dryland agriculture to acclimatize to future climatic conditions in this region (38). Our results also reinforce the need to utilize efficient crop production approaches such as appropriate crop selection and better cultivars (39) while minimizing the stress on groundwater resources in the United States (40, 41).

**Fig. 2.**
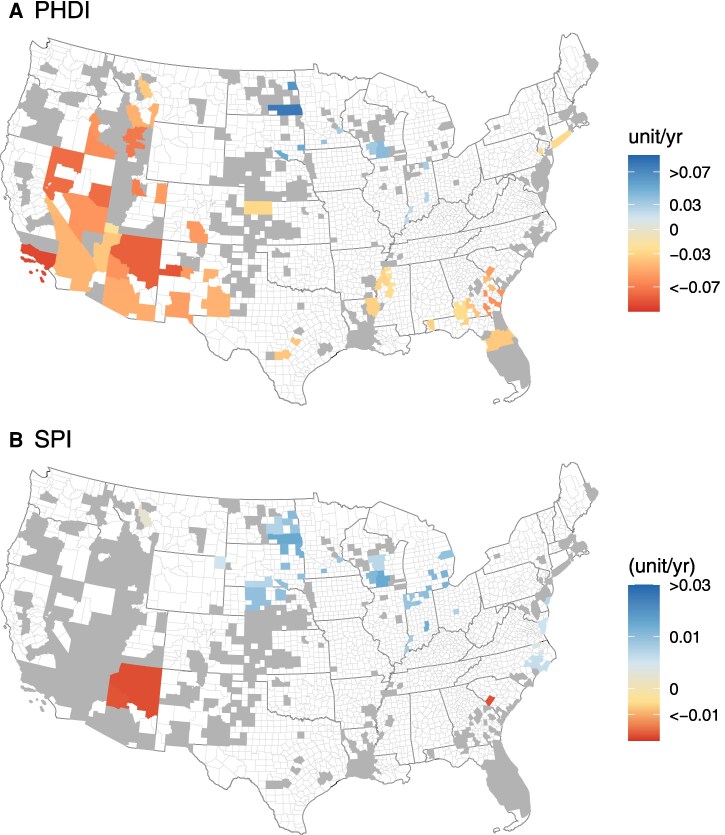
Temporal trends in Palmer drought hydrological index (A) and standard precipitation index (SPI)-12 (B), during 1970–2018. The color gradients represent the rate of change (i.e. sen slopes), and gray colored polygons represent no significant (*P* > 0.1) change in county-scale trends. The light and dark gray color polygons represent county and state boundaries of the United States, respectively.

Trend analysis of drought indices shows significant (*P* < 0.1) trends only in limited regions (<30% counties) during four decades (1970–2018), and the spatial patterns of significance varied between drought indices and the regions (Fig. 2). For example, the standard precipitation index-12 (SPI) shows no significant trend in the western United States, whereas the PHDI shows significant drying in the western United States (Fig. 2). Both drought indices reveal variable wetting trends in the corn belt region. Such differences in temporal trends between both drought indices are commonly observed and have been attributed to how each index is computed and what it represents (42, 43). For example, the SPI is based on precipitation anomalies and is much more temporally heterogeneous than the water balance derived Palmer index (42). Together, these spatiotemporal trends across counties underscore the need to utilize a combination of indices to unravel the drought patterns at the CONUS scale.

### Causal attribution of groundwater level patterns to crop production and drought

**Fig. 3.**
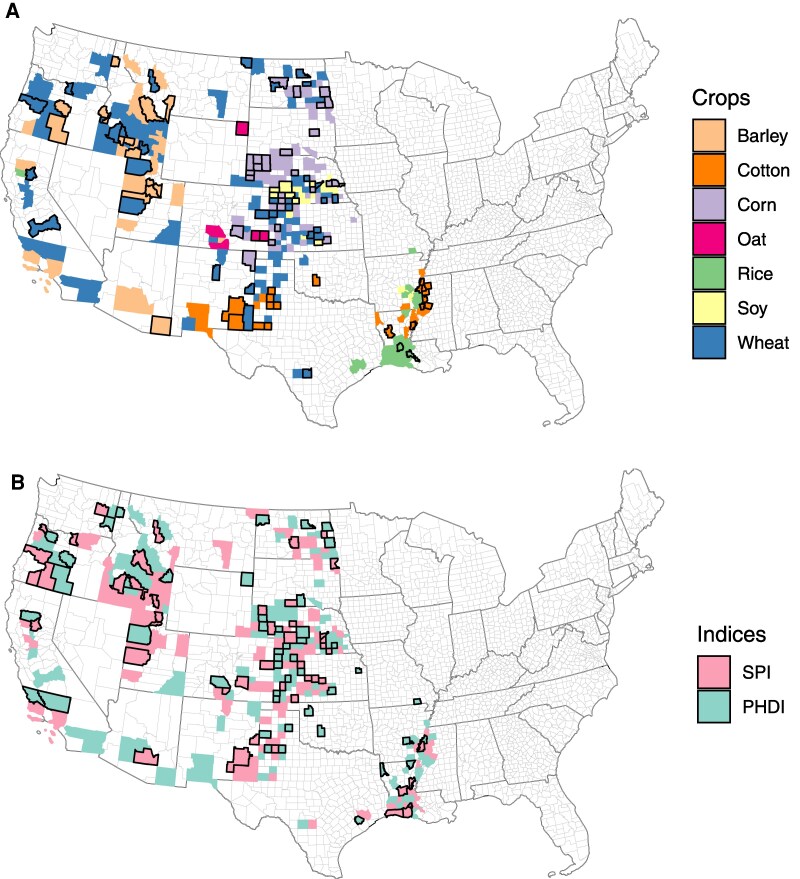
The causal influence of crop production (A) and drought (B) on groundwater levels across groundwater-irrigation-dominated counties of the United States. The unique color represents counties where the causal influence of each crop or drought index on groundwater levels is assessed, and the county boundaries with black outlines represent counties where we found significant (*P* < 0.1) causal influences. The light and dark gray color polygons represent county and state boundaries of the United States, respectively. SPI, Standard Precipitation Index-12; PHDI, Palmar Hydrological Drought Index.

**Fig. 4.**
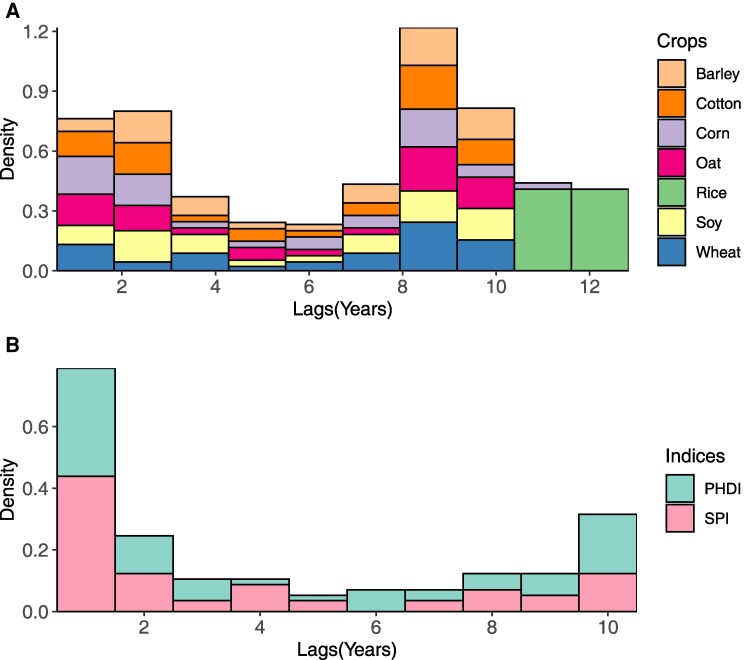
The distributions of the memory effect for the significant (*P* < 0.1) causal relationships between groundwater level and crop production (A), and groundwater level and drought (B), across groundwater-irrigation-dominated counties of the United States.

Using empirical observations and a causality-based attribution approach, we quantify the extent of the impact of drought and crop-specific productions in space and time on groundwater levels (Figs. 3 and 4). Not surprisingly, most of these counties correspond to some of the most groundwater-stressed regions of the United States (e.g. High Plains in the West). Further, the estimated memory effect in causal relationships demonstrates that the impact of crop production and drought on groundwater may last much longer (1–12 years) than often expected (Fig. 4).

### Crop production

We find a significant (*P* < 0.1) causal influence of crop production on groundwater levels in about one-third of the total groundwater-irrigation-dominated counties (∼300), whereas the extent of the effect varies with the crop type (Fig. 3, Table S1). The causal relationships are commonly detected between groundwater levels and cotton (42%, *n* = 18 counties), barley (23%, *n* = 15 counties), and wheat (17%, *n* = 39 counties) production. We further identify counties that show both causality and declining groundwater levels for individual crops (Table S1). Overall, there are 14% (*n* = 45 out of ∼300) counties where significant (*P* < 0.1) causality corresponded to significant (*P* < 0.1) decline in groundwater levels. For individual crops, groundwater levels have been significantly declining (*P* < 0.1) in 37–100% (Table S1, 3rd column) of the counties where causal influences are detected, implying that crop production can “cause” remarkable decline in groundwater levels. These findings agree with a global-scale study that attributed groundwater decline in the United States to the production of cotton, wheat, rice, and corn (15).

For a subset of counties where significant causal relationships are detected (Fig. 3), we quantify the memory effect of crop production on groundwater levels (Fig. 4). We note a wide variability (range: 1–12 years) in the memory effect of crop production on groundwater levels. The memory effect highlights that crop production can influence groundwater levels beyond the annual crop production cycle. These findings are not surprising as groundwater withdrawal to support crop production from the past years dictates the groundwater availability in the future. However, it is remarkable that the extent of impact could last up to a decade. We hypothesize the heterogeneity in the memory effect across crops and associated counties could be attributed to the variability in aquifer properties, climate, and water management. For example, some of the largest memory effects are estimated in the Columbia Plateau basaltic-rock aquifers (median: 10 years), Snake River (median: 10 years), and Coastal lowlands aquifer system (median: 9 years) that have small recharge rates and are located in an arid climate. Understanding the memory impact of crop production can inform farmers to adjust pumping or withdrawal rates to minimize their long-lasting impact on groundwater levels. Overall, our work underlines the circular causality between groundwater and crop production, where both entities are dependent on each other at different time scales. Our work is one of only a few studies to quantify the long-lasting impact of crop production on groundwater, and thus, emphasizes the relevance of accounting for historic crop estimates when managing for future groundwater (18).

### Drought

We find a significant (*P* < 0.1) causal influence of drought on groundwater levels in about 20% (SPI = 21%; *n* = 66 counties; PHDI = 20%; *n* = 62 counties) of irrigation-dominated counties (Fig. 3). Due to inherent differences between drought indices, it is not surprising that there are only 11% (*n* = 35 out of ∼300) counties where we detect the impact of both drought indices on groundwater levels. These findings indicate that the significant (*P* < 0.1) causal relationships noted are unique to the region and the drought index. Despite this spatial heterogeneity in causal relationships between drought indices and groundwater level, few consistent patterns emerge. For example, both drought indices reveal the widespread impact of drought on groundwater levels in the High Plains region in the West and the rice-producing region along the Mississippi River in Louisiana, United States (Fig. 3). Droughts may have further contributed to the groundwater scarcity problem in regions that are stressed due to over-exploitation of the aquifer for crop production. However, such regions are limited (<7%; *n* ∼ 20 counties) where we detected the significant (*P* < 0.1) causal influence of both crop production and drought on groundwater levels. These findings further complement other regional studies that have reported the impact of both crop production and drought on groundwater levels in some of the most stressed aquifers of the United States (36).

We find that the memory effect of drought on groundwater levels varied widely among counties ranging from 1 to 10 years (SPI, median: 2 years; PHDI, median: 3 years) (Fig. 4), emphasizing the extent of potential adverse impacts of droughts on groundwater. Not surprisingly, the memory effect for both indices in the same counties is almost the same (<3 years), where significant (*P* < 0.1) causality is detected for both drought indices. These findings suggest that the wide variation in memory effect across counties can be attributed to a combination of factors such as local climatic conditions, large-scale climate oscillations, physical properties of aquifers (transmissivity, hydraulic conductivity), and water management decisions that may influence groundwater levels. Our findings concur with a recent study, suggesting that the extent of droughts on groundwater levels could last between 1 and 14 years across pristine aquifers of the United States (25). Indeed, our estimated memory effects for drought fall within a similar range as previously (25) in pristine regions, but with much longer mean memory effects (mean: 4 years). Together, these findings imply that human interventions and water management may prolong the impact of drought on groundwater levels in agricultural-dominated regions. For example, it is a common practice to utilize more and deeper wells to fulfill the irrigation demand and offset the impact of drought on crop production in the United States and Asia (8, 10, 44). Such water management practices may result in a long-lasting impact of drought on groundwater and further increase the region's vulnerability to future droughts (45). However, more work is needed to establish and compare the long-lasting impact of drought on groundwater levels in pristine and managed regions of the United States.

### Sensitivity analyses, limitations, and implications

The sensitivity analysis of aquifer types (Figs. S2 and S3; SI Methods) shows that for unconfined aquifers (*n* = 112 counties), significant (*P* < 0.1) causality is detected for PHDI (26%, *n* = 30), SPI (17%; *n* = 20), and all crops (42%; *n* = 48 counties) supporting our earlier findings (Fig. 3). For confined aquifers, albeit with a much smaller sample size (*n* = 49 counties), we found significant (*P* < 0.1) causal relationships between groundwater levels and PHDI (30%; *n* = 15), SPI (22%; *n* = 11 counties), and all crops (18%; *n* = 9 counties). The findings from confined aquifers partially support our earlier analysis, but we acknowledge that generalization is difficult due to the small number of counties. Overall, this particular sensitivity analysis complements our earlier findings and partly addresses one of the challenges of aggregating observations at a county scale. Aggregating observations from point to regional scales often leads to the coupling of time series where only the most dominant patterns emerge and the short-lived and the local patterns are lost. It is possible that our work may have missed the local heterogeneity in groundwater patterns due to paucity of long-term groundwater observations and only underscores the regional scale patterns. Understanding the local groundwater pattern is essential, but the regional scale patterns are considered critical for the policy implications and better management of hydrologic systems (46, 47). Another key potential driver not included in our analysis is county-scale groundwater usage for irrigation available at 5-year intervals across CONUS (48). Due to a small (*n* < 12) sample size of groundwater usage data, we could not include this driver in our main analysis. However, because it is an important driver, we used linear interpolation to gap-fill the groundwater usage data and tested the role of groundwater withdrawal on groundwater patterns (SI; Methods). We find the significant (*P* < 0.1) causal influence of groundwater withdrawal on groundwater levels in 25% of the counties (*n* = 67 out of 273 counties; Fig. S4). Not surprisingly, we see a remarkable, significant (*P* < 0.1) causal influences in the High Plains aquifer in the West, a region that has experienced extensive groundwater withdrawal in the past three decades. These results need to be interpreted with caution as gap filling groundwater usage data are challenging due to the influence of droughts and change in water management practices on groundwater withdrawals. Overall, our work alludes to the data challenges we continue to face in understanding groundwater and food linkages at large spatial scales and indicates the need to collect high spatiotemporal observations to better manage these commodities in the future (22).

Our work highlights the causal linkages between widely grown field crops on groundwater levels across groundwater-irrigation-dominated counties. Based on the crop acreage data, the select crops are among the dominant (top 5) crops in most counties studied but are not necessarily dominant in all the counties. Consequently, our analysis may not have explored the causal linkages between the most dominant crop and groundwater level for some counties. If this is the case, it is possible that we may likely not have found statistically significant (*P* < 0.1) causal relationships for those counties, and our causal attribution to crop production is underestimated and conservative. Some counties in California have experienced a gradual transition from field crops to tree nuts, which are considered water-intensive commodities. Therefore, we tested the sensitivity of tree nuts production to groundwater levels in the counties where nuts are among the dominant (top five) crops based on Cropscape datasets (SI; Methods). We find a significant (*P* < 0.1) causal influence of almonds (*n* = 3 out of 6 counties) and pistachios (*n* = 1 out of 1 county) on groundwater levels (Fig. S5), while no significant (*P* > 0.1) causal influence is detected for walnut (*n* = 4 counties). Even though the sample size of this analysis is small, our findings highlight the likely role of tree nuts production in influencing groundwater levels. Future work is needed to expand on utilizing crop datasets from nearly 100 additional crops while accounting for seasonal variations in crop patterns and exploring their causal influence on groundwater levels across agricultural-dominated regions of the United States.

Using the gridded SPI data (SI; Methods), we find the causal impact of drought on groundwater level in 19% (*n* = 61) of counties (Fig. S6). So, our findings from the sensitivity analysis of SPI index support our initial results showing a significant (*P* < 0.1) causal influence of drought on groundwater levels in 20% (*n* = 66) counties (Fig. 3). While the number of counties where causal influence is detected between both SPI products is similar, the spatial patterns of causal significance are not, as 19 out of ∼60 counties where both indices show significant (*P* < 0.1) causality. Thus, the sensitivity analysis highlights the need to utilize multiple products to assess the impact of drought on groundwater levels, depending on the spatial scale of interest.

To summarize, we show declining groundwater levels and increasing crop production as one of the dominant trends across the United States (Fig. S1). Further, we noted that declining groundwater levels corresponded to declining or no change (i.e. stagnation) in crop production for several agricultural regions of the United States (e.g. the High Plains in the West). For these regions, these results imply that we continue to extract groundwater at the same rate (49), as in the past, without any substantial change in crop production. Concurrently, to better manage groundwater in the near future, our work underscores an urgent need to develop long-term irrigation datasets for individual crops at a finer spatial resolution across CONUS. The causality models developed here allowed us to use empirical observations to highlight that the memory effect of crop production or drought on groundwater levels could last for more than a decade. Unraveling the memory impact of crop production in advance can inform farmers to adjust withdrawal rates to minimize their long-lasting impact on groundwater levels. Additionally, our work documents the spatial distribution of vulnerable agricultural regions that require efficient agricultural and water management practices in the face of changing climate and/or declining water availability to meet future food and water demands. These findings indicate a pressing need to develop drought mitigation efforts (50–53), such as implementing managed aquifer recharge, improving irrigation efficiency, and planting less water-intensive crops to minimize the long-lasting impact of drought on groundwater.

## Materials and methods

### Site selection and datasets

#### Site selection

We used long-term averages of groundwater-irrigation withdrawals and irrigated agricultural areas to identify groundwater-irrigation-dominated counties across the United States provided by the USGS National Water-Use Information Program (48, 54) at 5-year intervals (1995–2015). In this analysis, we included only counties with higher groundwater usage for irrigation (>50th percentile, i.e. 0.47 Mgal/day) and greater irrigated agricultural areas (>50th percentile, i.e. 1,440 acres). Consequently, we included a total of 1,269 counties across the United States in this study. A subset of these counties was used for trend and attribution analyses, depending on the availability of groundwater, crop production, and drought datasets.

#### Datasets

Groundwater levels, defined as “depth to water level from the surface,” were obtained from USGS National Water Information System (54). Utilizing the wells that fulfilled selection criteria (SI; Materials), we computed regional median groundwater levels over the well locations within each study county (∼550) annually from 1970 to 2018 (SI; Materials). To understand the crop-specific withdrawal impact on groundwater levels, we used long-term estimates of irrigated crop production (kg/acre) for seven field crops from the US Department of Agriculture, National Agricultural Statistics Service (SI; Materials). We focused on 384 counties (wheat), 243 (corn), 105 (cotton), 132 (barley), 134 (soybean), 29 (oat), and 71 counties (rice) out of 1,269 counties (SI; Materials). For drought products, we obtained time series of two commonly used drought indices, PHDI and the SPI, from the National Climatic Data Center during 1970–2018 (55). Both drought products are available at climate division spatial resolution and available across CONUS (SI; Materials).

### Statistical analysis

#### Trend analysis

We used the Mann–Kendall test, a rank-based and nonparametric approach to estimate monotonic temporal trends in median groundwater levels, crop productions, and drought indices for each county (56). We computed sen slopes to quantify the annual rate of change in each time series for an individual county (SI; Methods).

#### Attribution analysis

We used Granger Causality modeling (57) to attribute groundwater level patterns to crop production and climate at the county scale across the United States. This approach allowed us to quantify the relative influence of crop production and drought on groundwater levels, but it does not explicitly indicate directionality in relationships between both drivers and groundwater levels. The analysis was implemented on the subset of counties (*n* ∼ 300) where groundwater levels (Fig. S7) along with crop production and drought indices were available (SI; Methods). We tested the hypothesis that drought indices or crop production can “cause” groundwater level changes within each county. Further, to quantify memory effect for the subset of counties where we detected significant (*P* < 0.1) causality, we tracked the maximum significant (*P* < 0.1) lags (i.e. max number of years) in the relationship (SI; Methods).

## Supplementary Material

pgaf129_Supplementary_Data

## Data Availability

All datasets used in the study are publicly available, and citations have been provided in the references.
