# Supplementary material for: Unraveling the causal influences of drought and crop production on groundwater levels across the contiguous United States
Source: PNAS Nexus. 2025 Apr 28;4(5):pgaf129. doi: 10.1093/pnasnexus/pgaf129 (PMC12056729; doi:10.1093/pnasnexus/pgaf129)
Supplement: pgaf129_Supplementary_Data [file pgaf129_supplementary_data.pdf]

Supplementary information for

Unraveling the causal influences of drought and crop production on groundwater levels across the contiguous United States

Nitin K. Singh[a*], Sheila M. Saia[b,c], Ruchi Bhattacharya[d], Hoori Ajami[e], David M. Borrok[f]

Author Affiliations

[a]Crop, Soil, and Environmental Sciences, Auburn University, Auburn, AL, 36849, USA
[b]Tetra Tech, Research Triangle Park, Durham, NC, 27703 USA
[c]Biological and Agricultural Engineering, North Carolina State University, Raleigh, NC, 27695, USA
[d]Biological, Geological and Environmental Sciences, Cleveland State University, Cleveland, OH, 44115, USA
[e]Department of Environmental Sciences, University of California Riverside, Riverside, CA, 92521, USA
[f]College of Engineering and Computing, Missouri University of Science and Technology, Rolla, MO, 65409, USA

*Corresponding author: Nitin K. Singh; **Email:** nitin.singh@auburn.edu

Mailing address of corresponding author: Crop, Soil and Environmental Sciences, Auburn University, 350 South College St, Auburn, AL 36849.

Author Contributions:

Nitin K Singh
Conceptualization
Data curation
Formal analysis
Visualization
Writing-original draft

Sheila M Saia
Data curation
Investigation
Visualization
Writing-review and editing

Ruchi Bhattacharya
Data curation
Investigation
Visualization
Writing-review and editing

Hoori Ajami

Investigation
Writing-review and editing
David M Borrok
Investigation
Writing-review and editing

Competing Interest Statement: The authors declare no competing interests.

Classification: Environmental Sciences (Physical Sciences and Engineering)

Keywords: Water Sustainability, Food Security, Groundwater Decline, Causality Modeling, Drought

This PDF file includes:

Text Extended Materials and Methods
Figures S1-S7
Table S1
References

Supporting Information Text

Materials

Datasets

Following the criteria (9), we downloaded groundwater levels for wells with more than 100 observations during 1970-2018. We selected the groundwater wells that have a minimum of 20 years of record with at least one or more observations per year (25th percentile: 8 observations/year; 50th percentile: 20 observations/year; 75th percentile: 62 observations/year; and 95th percentile: 250 observations/year). Further, we computed regional median groundwater levels over the well locations within each study county annually from 1970 to 2018. We focused on counties where median groundwater levels were available for at least 20 years between 1970 and 2018. This criterion led to the selection of ~550 counties (out of 1,269 counties) with long-term median groundwater levels. Utilizing the delineated boundaries of major aquifers provided by USGS, we aggregated the changes in groundwater levels for each major aquifer that overlapped with the selected groundwater irrigation-dominated counties.

To understand the crop-specific withdrawal impact on groundwater levels, we focused on seven field crops, including wheat, corn, soybean, rice, cotton, barley, and oat, that represent approximately 60% of the acreage area of the US based on the long-term annual means (2008-2018; (58)) and have some of the highest irrigation water usage in the United States (14). Given that the demand for such field crops is likely to increase in the future, it is vital to understand the likely footprint of these field crops on groundwater levels. We ranked the field crops based on the long-term (2008-2018) mean annual acreage and extracted the top three dominant crops for each county. However, annual crop production datasets were not consistently available for all sites, so we focused on counties where the production estimates were available for at least 20 years between 1970 and 2018. These criteria led to the selection of 384 counties (wheat), 243 (corn), 105 (cotton), 132 (barley), 134 (soybean), 29 (oat), and 71 counties (rice) out of 1269 counties.

To understand the causal relationship between drought and groundwater levels, we selected two commonly used drought indices, Palmer Drought Hydrological Index (PHDI) and the Standard Precipitation Index (SPI), which are often used in groundwater studies (28, 29, 59, 60).

These two indices represent different levels of sensitivity to drought (59, 61). The PDHI is derived from monthly water balance (59, 62), whereas SPI is based on precipitation anomalies over a range of temporal scales (63). It is worth acknowledging that the SPI over a longer duration (e.g., 12 months) is considered more relevant for understanding the impact of drought on groundwater levels (59, 64). Thus, we obtained the time series of PHDI and the SPI-12 months (here onward SPI) from the National Climatic Data Center (NCDC) during 1970-2018 (55). Both drought products are available at the climate-division scale across CONUS.

Methods

Trend Analysis

We used the Mann-Kendall test to estimate monotonic temporal trends in median groundwater levels, crop productions, and drought indices for each county (56). We computed temporal trends in annual median groundwater levels for each study county. Likewise, for each crop, we estimated the temporal trend in annual crop production at the county level. Trend detection approaches can lead to spurious trends due to autocorrelation in datasets (65, 66). We implemented a commonly used variance correction approach to address autocorrelation in time series (65). We used sen slopes to compute the rate of change in annual median groundwater levels and drought indices for an individual county. Similarly, for every crop, we used the sen slope to compute the rate of change in annual crop production within each county. To further explore how trajectories of groundwater level and crop production have co-evolved, we compared trends of annual median groundwater levels and annual crop production at the county scale and focused our analysis on a subset of counties where both groundwater and crop production observations were available for the same duration.

Attribution Analysis

We used Granger Causality modeling (57) to attribute groundwater level patterns to crop production and climate at the county scale across the United States. Since the inception of Granger causality in 1969, it has been widely used in climate change attribution (31, 33, 67, 68) and recently in identifying causal relationships in food-water nexus (10, 69). Granger proposed a

bivariate model with two non-stationary time series (X and Y) that generally follows equation (1) (57). Time series X is set to “cause” Y if the predictability of Y increases by including all the information available except the current value of X.

$$Y_t = \sum_{j=1}^{m} u_j Y_{t-j} + \sum_{j=1}^{m} k_j X_{t-j} + \varepsilon_t \qquad (1)$$

where X and Y are two stationary time series; u and k are coefficients; ε is white noise. As noted in the equation 1, Granger Causality assumes that the relationship between exogenous and endogenous variable is not changing over time. We implemented causality modeling in the following steps (10, 70, 71). First, we conducted the Phillips-Perron test to check the stationarity of both endogenous and exogenous variables for each county (72). If the time series is non-stationary, we detrended the time series using differencing (70). Second, we used this non-stationary time series to fit the vector autoregressive (VAR) model to predict endogenous (i.e., groundwater level) as a function of each exogenous variable (i.e., crop production or drought) at the county level. Lastly, we used an F test to evaluate the null hypothesis: the exogenous variable does not ‘cause’ groundwater levels to change over time. We repeated these steps for each of the seven crops and two drought indices separately for an individual county, resulting in more than 900 VAR models. The VAR model also allows us to explore if there is a memory effect in the relationship between endogenous and exogenous variables (73). For the subset of counties where we detected significant ($P < 0.1$) causality, we tracked the maximum significant ($P < 0.1$) lags (i.e., max number of years) the relationship can last in the VAR model. Causality modeling was conducted where we had at-least 20 years of observations for groundwater and crop estimates. Fig S7 summarizes the total number of observations and the numbers of wells within each county used for causality modeling during the study period.

Causality-based driver attribution analysis has many advantages over complex continental-scale water budgeting or hydrological models. The statistical model is not computationally intensive and is relatively less cumbersome to build than a continental-scale physically-based hydrological model. Hydrological models at such large spatial scales have large uncertainties and

assumptions due to scores of parameters. However, causality attribution is entirely based on the availability of long-term empirical observations. Causality analysis is ultimately a statistical modeling approach and has limitations too. For example, causality models are based on statistical relationships between exogenous and endogenous variables and not on physical processes. That said, statistical models have been widely used to explore the food-water-climate nexus (6, 74, 75) and findings are still bound by the input data provided and the usage of statistical significance levels (P value). It is important to acknowledge that like many other statistical approaches, Granger causality does not account for interaction effects of other plausible drivers on the response variable. Lastly, we acknowledge that it would have been ideal to conduct attribution and trend analyses at the aquifer scale but due to a lack of crop production data at the same spatial scale, the analyses are executed at the county scale.

Sensitivity Analysis

While the major goal of the study is to explore the impact of drought and crop production, a few factors and methods-related limitations may affect our findings but could not be accounted for due to the lack of data. We conducted sensitivity analysis for two aspects of our methods. First, our work utilizes groundwater levels from several wells (point-scale) to estimate county-scale groundwater levels. While the aggregation exercise undertaken here is not new and has been used before (19), it can result in integrating groundwater levels from diverse geological settings with differences in storage coefficients (e.g., unconfined versus confined aquifers), influencing spatiotemporal patterns of groundwater storage (76, 77). To test the sensitivity of the aquifer type on causal relationships, we grouped the wells into unconfined and confined aquifers and re-conducted the causality modeling separately for each group. Another important driver that could not be incorporated in our analysis is the potential role of groundwater withdrawal for irrigation due to limited sample size ($n<12$). Because it is an important driver, we explored the role of groundwater usage for irrigation on groundwater patterns. We obtained the groundwater usage data for irrigation at 5-year intervals between 1985 and 2015 for our study counties from USGS. Counties with missing or no data for any of the 5 years were excluded from our analysis,

resulting in about 273 counties with no missing data at 5-year intervals. We used linear interpolation to fill gaps between five-year durations to construct annual time series. This approach led to an adequate sample size at a county level to conduct causality modeling.

The quantification of drought is partly sensitive to the quality of the source data (78). Studies recommend confirming drought-related findings based on different source datasets to address this concern. Here, we test the sensitivity of the causality analysis to one of the drought products used (i.e., SPI) in the study. We re-examine the causal influence of drought on groundwater levels using global gridded SPI datasets (1x1 degree, spatial resolution) available between 1949-2012 through NCAR (79). The gridded SPI has a different spatial resolution and the underlying source precipitation than the original climate-division scale SPI data (55) available across CONUS.

Lastly, some counties in California transitioned from field crops to tree nuts, which could have influenced the groundwater levels in those counties. Therefore, we conducted causality modeling to test the sensitivity of tree nuts to groundwater levels. It is important to highlight that tree nuts-related datasets are not readily available through the USDA National Agriculture Statistics Services API (like other field crops) but must be downloaded individually for each nut annually (1970-2018) from the National Agriculture Statistics Services website. Based on the Cropscape datasets (58), we find seven counties in which almonds, pistachios, or walnuts are among the dominant crops (top five). So, we focused on these seven counties for the causality modeling.

Table S1. The percentage of counties showing significant (P < 0.1) causality and declining groundwater (GW) levels

Crops	Causality Detected %(n‡)	Causality with Significant GW Decline % (n‡)	Causality with Significant GW Decline/Causality Detected %*
Barley	23(15)	10(7)	46
Rice	5(2)	5(2)	100
Cotton	42(18)	23(10)	55
Corn	20(27)	7(10)	37
Wheat	17(39)	7(16)	41
Oat	21(3)	14(2)	66
Soybean	11(7)	6(4)	57

‡n represents the sample size for each percentage value; *Third column is equal to the ratio of the first and second columns, and it represents the fraction of counties with significant (P < 0.1) causal relationships where groundwater level has declined.

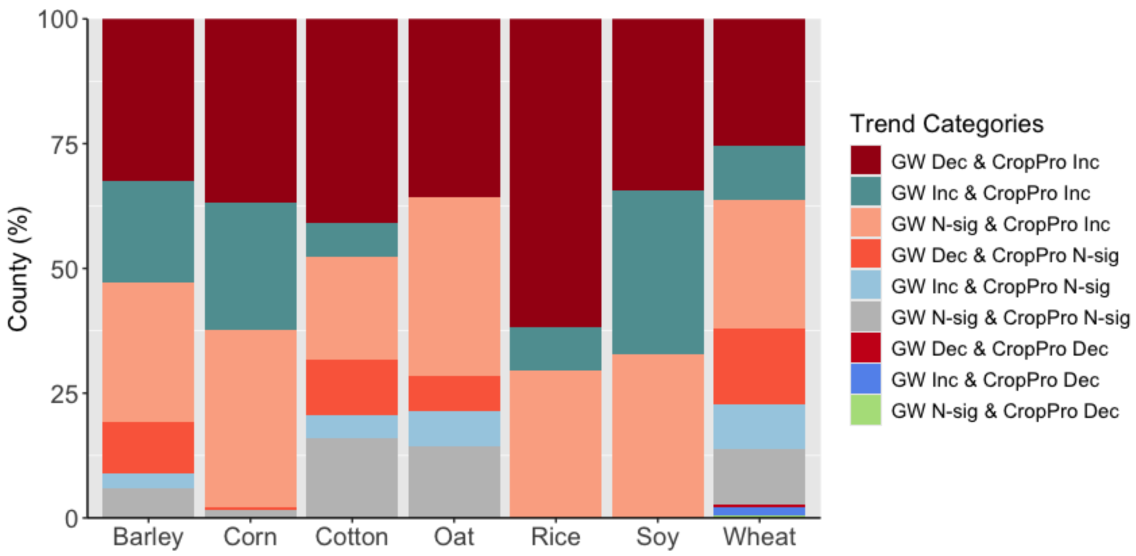

Fig S1. A comparison of temporal trends between groundwater levels (GW) and crop production (CropProd) at the county scale. Declining groundwater levels with increasing crop production is one of the dominant trends across all crops. Abbreviations: groundwater (GW), declining trend (Dec), increasing trend (Inc), non-significant trend (N-sig), crop production (CropProd).

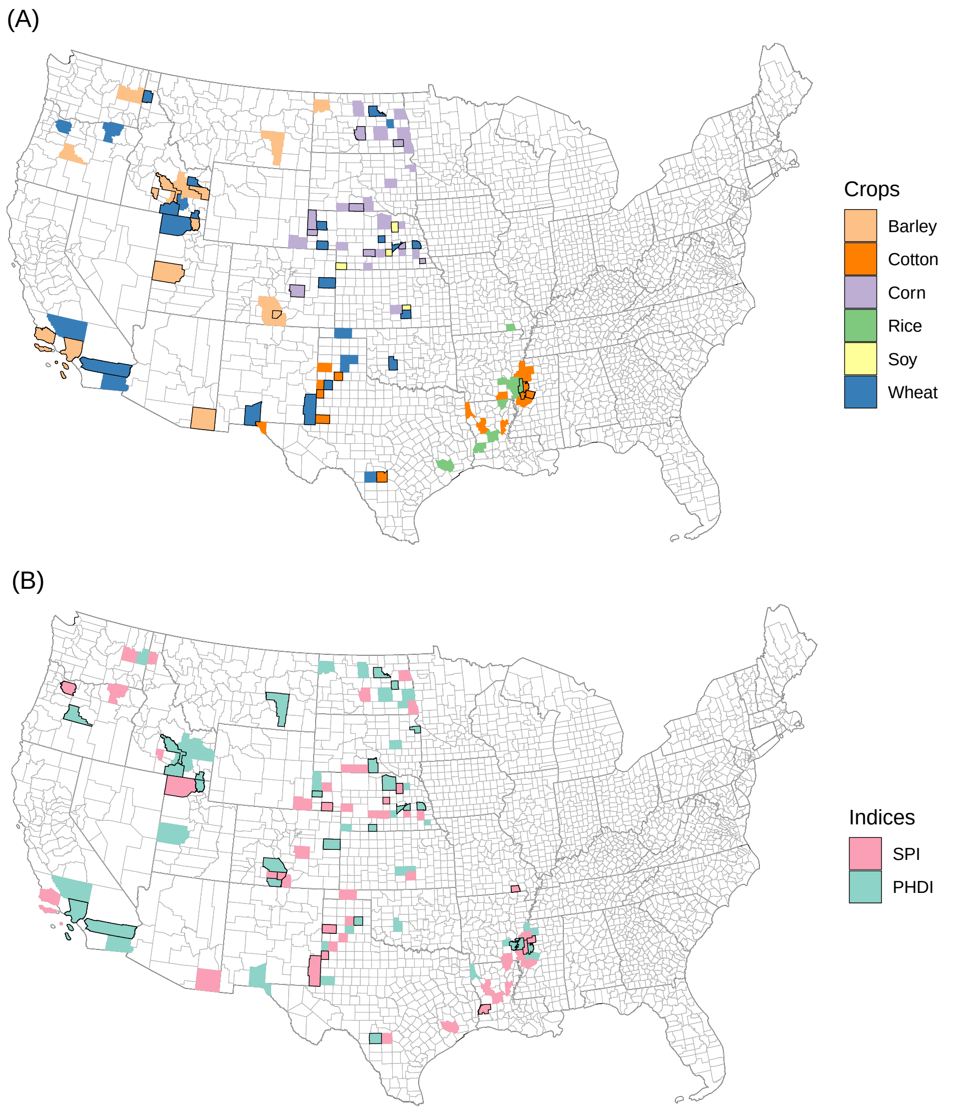

Fig S2. The causal influence of crop production A) and drought B) on groundwater levels in unconfined aquifers across groundwater irrigation-dominated counties of the US. The unique color represents counties where the causal influence of each crop or drought index on groundwater levels is assessed, and the county boundaries with black outlines represent counties where we found significant ($P < 0.1$) causal influences. The light and dark gray color polygons represent county and state boundaries of the US, respectively. Abbreviations- Standard Precipitation Index (SPI), Palmar Hydrological Drought Index (PHDI).

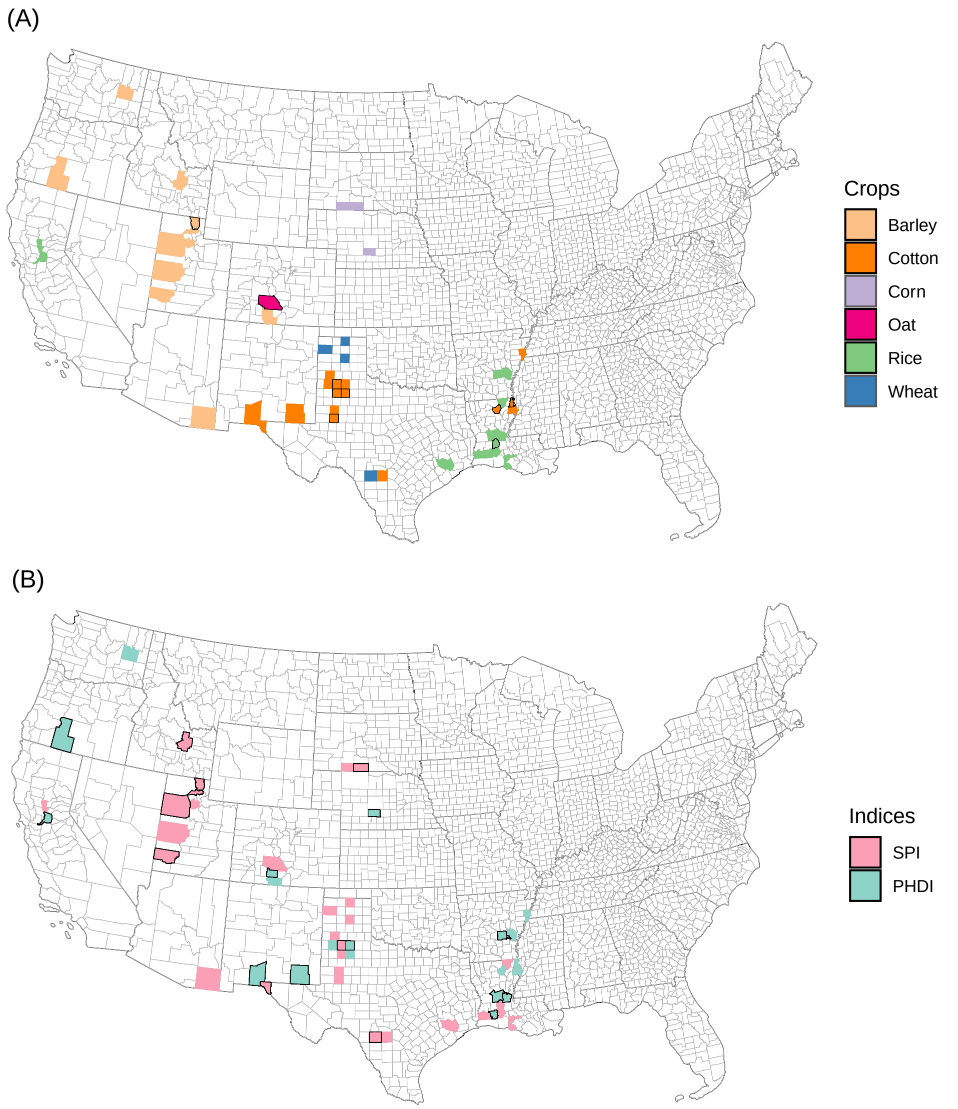

Fig S3. The causal influence of crop production A) and drought B) on groundwater levels in confined aquifers across groundwater irrigation-dominated counties of the US. The unique color represents counties where the causal influence of each crop or drought index on groundwater levels is assessed, and the county boundaries with black outlines represent counties where we found significant ($P < 0.1$) causal influences. The light and dark gray color polygons represent county and state boundaries of the US, respectively. Abbreviations- Standard Precipitation Index (SPI), Palmar Hydrological Drought Index (PHDI).

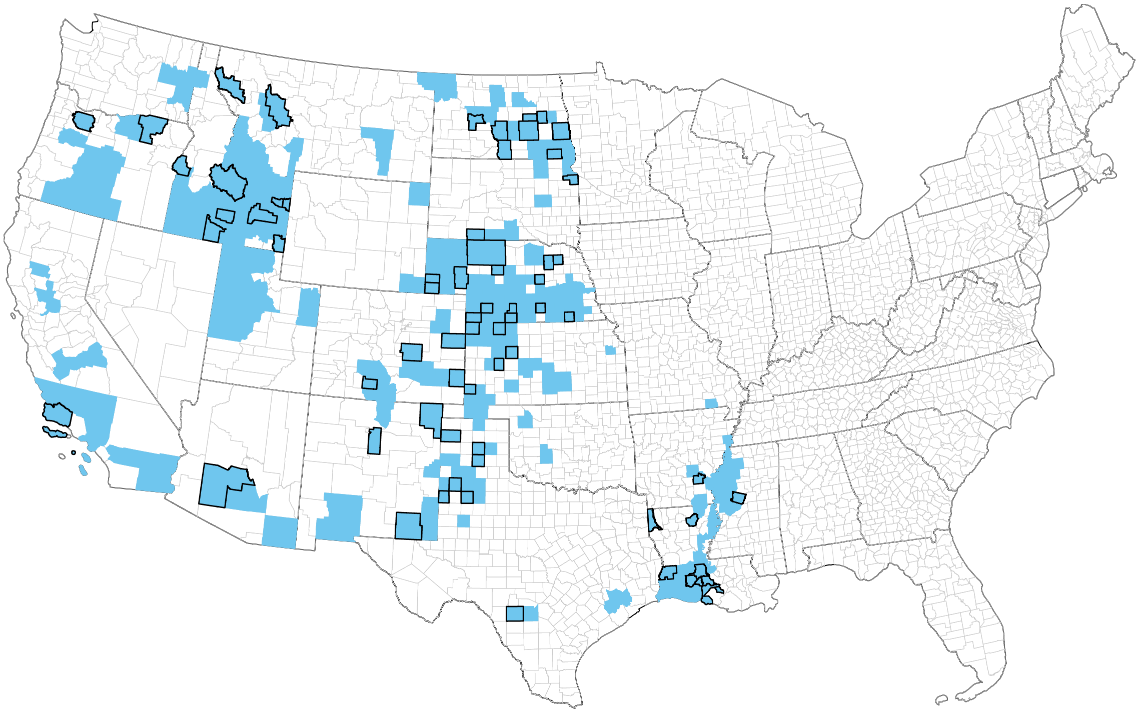

Fig S4. The causal influence of irrigation-groundwater withdrawal on groundwater levels across the United States. The light blue color represents counties where we assessed the causal influence of irrigation-related groundwater withdrawal on groundwater levels across groundwater irrigation-dominated counties of the US. The county boundaries with black outlines represent counties with significant ($P < 0.1$) causal influences. The light and dark gray color polygons represent county and state boundaries of the US, respectively.

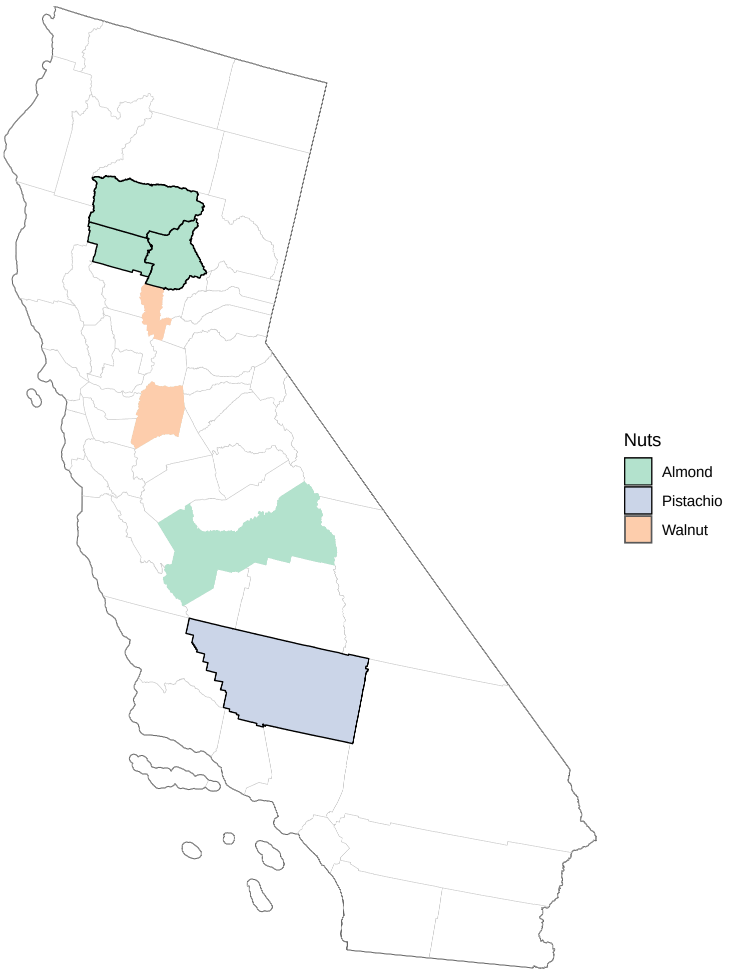

Fig S5. The causal influence of tree nuts (almond, pistachio and walnut) on groundwater levels in California (CA). The unique color represents counties where the causal influence of each nut on groundwater levels is assessed, and the county boundaries with black outlines represent counties with significant ($P < 0.1$) causal influences. The light gray color polygons represent the county boundaries of the CA, respectively.

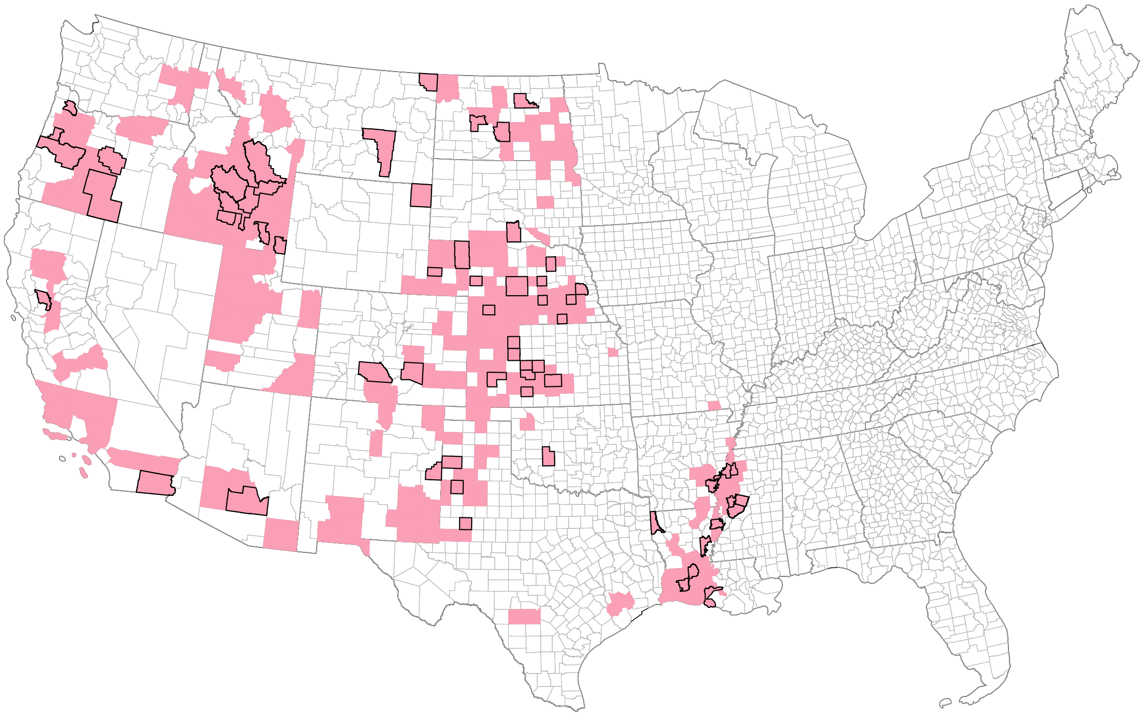

Fig S6. The causal influence of drought, derived from gridded SPI-12 datasets, on groundwater levels across groundwater irrigation-dominated counties of the US. The light pink color represents counties where we assessed the causal influence of SPI-12 on groundwater levels, and the county boundaries with black outlines represent counties where we found significant (P < 0.1) causal influences. The light and dark gray color polygons represent county and state boundaries of the US, respectively. Abbreviations- Standard Precipitation Index (SPI)

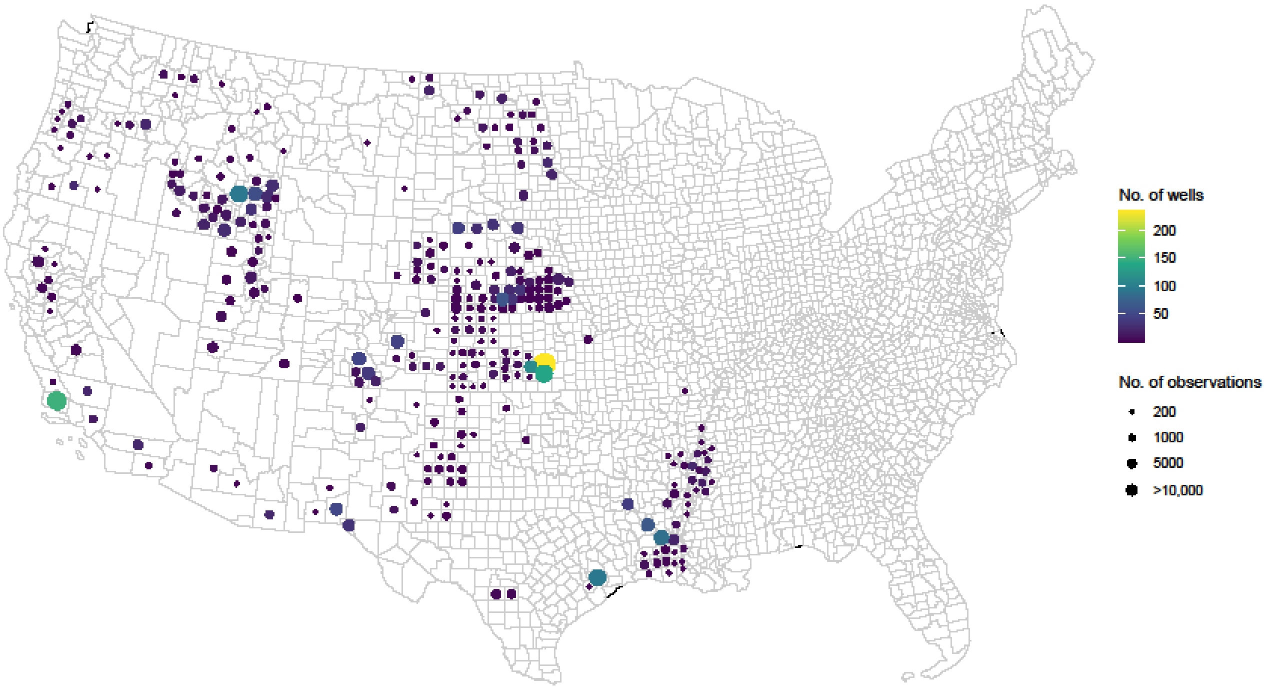

Fig S7. The number of wells and number of observations used in the causality modeling.

References

58. W. Han, Z. Yang, L. Di, R. Mueller, CropScape: A Web service based application for exploring and disseminating US conterminous geospatial cropland data products for decision support. Comput. Electron. Agric. 84, 111–123 (2012).

59. M. D. Svoboda, B. A. Fuchs, Handbook of Drought Indicators and Indices: Integrated Drought Management Programme (IDMP) (World Meteorological Organization, 2016).

60. M. Zhao, A. Geruo, I. Velicogna, J. S. Kimball, Satellite Observations of Regional Drought Severity in the Continental United States Using GRACE-Based Terrestrial Water Storage Changes. J. Clim. 30, 6297–6308 (2017).

61. R. R. Heim, A Review of Twentieth-Century Drought Indices Used in the United States. Bull. Am. Meteorol. Soc. 83, 1149–1166 (2002).

62. W. C. Palmer, Meteorological drought. US. Weather Bureau Res. Paper 45, 1–58 (1965).

63. T. Mckee, N. Doesken, J. Kleist, The relationship of drought frequency and duration to time scales. 17, 179–183 (1993).

64. A. Zargar, R. Sadiq, B. Naser, F. I. Khan, A review of drought indices. Environ. Rev. 19, 333–349 (2011).

65. K. H. Hamed, A. Ramachandra Rao, A modified Mann-Kendall trend test for autocorrelated data. J. Hydrol. (Amst.) 204, 182–196 (1998).

66. S. Yue, P. Pilon, B. Phinney, G. Cavadias, The influence of autocorrelation on the ability to detect trend in hydrological series. Hydrol. Process. 16, 1807–1829 (2002).

67. A. Attanasio, A. Pasini, U. Triacca, A contribution to attribution of recent global warming by out-of-sample Granger causality analysis. Atmos. Sci. Lett. 13, 67–72 (2012).

68. L. Olsson, H. Thorén, D. Harnesk, J. Persson, Ethics of probabilistic extreme event attribution in climate change science: A critique. Earths Future 10 (2022).

69. T. Deryugina, M. Konar, Impacts of crop insurance on water withdrawals for irrigation. Adv. Water Resour. 110, 437–444 (2017).

70. D. D. Zhang, et al., The causality analysis of climate change and large-scale human crisis. Proc. Natl. Acad. Sci. U. S. A. 108, 17296–17301 (2011).

71. B. Pfaff, VAR, SVAR and SVEC Models: Implementation WithinRPackagevars. J. Stat. Softw. 27, 1–32 (2008).

72. P. C. B. Phillips, P. Perron, Testing for a unit root in time series regression. Biometrika 75, 335 (1988).

73. L. Del Rio Amador, S. Lovejoy, Long-range forecasting as a past value problem: Untangling correlations and causality with scaling. Geophys. Res. Lett. 48, e2020GL092147 (2021).

74. D. B. Lobell, M. B. Burke, On the use of statistical models to predict crop yield responses to climate change. Agric. For. Meteorol. 150, 1443–1452 (2010).

75. N. Bhattarai, et al., Warming temperatures exacerbate groundwater depletion rates in India. Sci. Adv. 9, eadi1401 (2023).

76. A. Rateb, et al., Comparison of groundwater storage changes from GRACE satellites with monitoring and modeling of major U.s. aquifers. Water Resour. Res. 56, e2020WR027556 (2020).

77. D. Zhang, X. Liu, C. T. Simmons, L. Zhang, Q. Zhang, Changes in groundwater levels across China from 2005 to 2016. J. Hydrol. (Amst.) 623, 129781 (2023).

78. K. E. Trenberth, et al., Global warming and changes in drought. Nat. Clim. Chang. 4, 17–22 (2014).

79. National Center for Atmospheric Research/University Corporation for Atmospheric Research, Standardized Precipitation Index (SPI) for Global Land Surface (1949-2012). UCAR/NCAR - Research Data Archive. https://doi.org/10.5065/D6086397. Deposited 19 December 2013.
